# Comparison of magnetic resonance enterography, capsule endoscopy and gastrointestinal radiography of children with small bowel Crohn’s disease

**DOI:** 10.3892/etm.2013.1113

**Published:** 2013-05-14

**Authors:** CAN LAI, HAI-CHUN ZHOU, MING MA, HONG-XI ZHANG, XUAN JIA

**Affiliations:** 1 Departments of Radiology, Zhejiang University School of Medicine, Hangzhou, Zhejiang 310000, P.R. China; 2 Gastroenterology, Children’s Hospital, Zhejiang University School of Medicine, Hangzhou, Zhejiang 310000, P.R. China

**Keywords:** magnetic resonance enterography, small bowel Crohn’s disease, video capsule endoscopy, conventional gastrointestinal radiography

## Abstract

The aim of this study was to compare magnetic resonance enterography (MRE) findings with those of video capsule endoscopy (VCE) or conventional gastrointestinal radiography (CGR) in pediatric patients with small bowel Crohn’s disease. A total of 55 cases of small bowel Crohn’s disease that were diagnosed through clinical, laboratory, surgical and histopathological findings were reviewed. Prior to the examination, children suspected of having other types of diseases of the small intestinal were identified. The pulse sequences included coronal T2-true-fast imaging with steady-state precession (TrueFISP) images, navigation axial and coronal T1-weighted images, T2-weighted fat-suppressed images and coronal fat-suppressed three-dimensional gradient-echo images, immediately followed by contrast-enhanced axial and coronal T1-weighted fat-suppressed images. Findings from MRE were compared with those of VCE (n=39) and CGR (n=37). MRE results exhibited a number of features characteristic to small bowel Crohn’s disease, including wall thickening, mesenteric fibrofatty changes and mesenteric vasculature changes. VCE, MRE and CGR demonstrated sensitivities of 94.6, 85.7 and 71.1% with specificities of 72.7, 70 and 40%; accuracies of 89.6, 82.2 and 61.1%; positive predictive values of 92.1, 90.9 and 59.6%; and negative predictive values of 80, 58.3 and 40%, respectively. VCE depicted mucosal pathologies missed by MRE in three patients. MRE revealed 83 extraenteric findings in 55 patients and CGR was able to show the dynamic evolution of the gastrointestinal function. MRE is a simple, safe, non-invasive and effective method for evaluating small bowel Crohn’s disease. VCE allows visualization and readily characterizes subtle mucosal lesions missed by MRE, whereas MRE yields additional mural, perienteric and extraenteric information. However, oral barium CGR utilizes radiation, which is not suitable for repeated use in children.

## Introduction

The small intestine is the longest gastrointestinal (GI) organ; it bends at a number of points and often overlaps. Clinical imaging and evaluation of the small bowel has always been challenging. Endoscopic techniques are usually limited to the most proximal and distal regions and thus, small bowel follow-through and conventional gastrointestinal radiography (CGR) have long been the primary methods in small bowel assessment. However, the diagnostic yield of these methods is low, particularly in detecting sources of obscure GI bleeding, early inflammatory changes and small mass lesions. The diagnostic accuracy of these methods is dependent on the examiner’s experience at a relatively high degree. Moreover, CGR, which is semi-invasive, entails exposure to ionizing radiation and may cause discomfort for a significant number of patients (1).

Magnetic resonance enterography (MRE) techniques are emerging modalities, which are increasingly supplanting conventional radiological methods in the evaluation of small bowel pathology, while providing vast extraluminal information (2). Magnetic resonance imaging (MRI) with fast scanning and three-dimensional post-processing abilities clearly reveals abnormalities of the wall and outside structure of the GI lumen. The good soft tissue contrast and three-dimensional imaging capabilities of MRE allow observation of the mucosa and analysis of changes of peripheral structures, which increases the information gained during the diagnostic imaging of GI diseases. MRE is more sensitive to pathological changes and is free of radiation, which makes it tremendously advantageous in the diagnosis and treatment evaluation of pediatric diseases of the small intestine (3).

The development of wireless video capsule endoscopy (VCE) has been widely considered to be a breakthrough for endoluminal visualization of the entire small bowel. VCE is a revolutionary diagnostic tool for small bowel diseases. Since it was introduced, VCE has become key to the diagnostic evaluation of obscure GI bleeding. VCE is appealing to patients and providers since it is a relatively non-invasive imaging modality, and the requirement to expand its diagnostic role continues to increase. The use of VCE in the diagnosis of Crohn’s disease and chronic diarrhea, and the potential of employing VCE as a cancer surveillance mechanism in patients with hereditary polyposis syndromes that may involve the small bowel are current topics of continuing further study. VCE has demonstrated an unprecedented high sensitivity in detecting sources of obscure bleeding and is widely established in the work-up algorithm of patients presenting with acute upper gastrointestinal hemorrhage (4). Additionally, VCE has shown promise in the evaluation of suspected Crohn’s disease and small bowel tumors (5). A number of studies have compared VCE with conventional enteroclysis; VCE has been compared with traditional diagnostic modalities, including small bowel series, enteroclysis, computed tomography (CT) and push enteroscopy (PE) (6–9). Small bowel VCE is the procedure of choice for evaluating obscure GI bleeding and is superior to radiographic procedures in detecting Crohn’s disease of the small bowel. However, data comparing MRE with VCE are limited to relatively small study cohorts in the assessment of small bowel diseases (10,11). We collated 55 cases of small bowel Crohn’s disease in the Children’s Hospital of Zhejiang University School of Medicine, during a 5-year period, each of which had clinical and pathological results. The aim of this study was to prospectively assess VCE, MRE and CGR in the diagnostic work-up of patients with a broad range of indications of suspected small bowel Crohn’s disease.

## Materials and methods

### General data

Retrospective analysis was performed on a total of 55 cases, which were collated in the Children’s Hospital of Zhejiang University School of Medicine from February 2008 to December 2012. Of these, 31 were male and 24 were female (age range, 12 months to 16 years). Clinical symptoms included abdominal pain, diarrhea, abdominal distension, fever, abdominal mass, melena and bloody stools. This study was conducted in accordance with the Declaration of Helsinki, with approval from the Ethics Committee of Zhejiang University. Written informed consent was obtained from all participants.

### MRE protocol

Patients to be examined by hydro-MRI of the small intestine were administered 2.5% isotonic mannitol (Shandong Weigao Pharmaceutical Co., Ltd., Shandong, China) orally every 15 min for 45–60 min prior to scanning. The oral contrast agent was administered three times (an average dose 45–60 min before scanning; a half dose 15–30 min before scanning; and a half dose 5 min before entering the MRI room), followed by an intravenous injection of 0.3 mg/kg anisodamine (Hangzhou Minsheng Pharmaceutical Group Co., Ltd., Hangzhou, China) to inhibit intestinal motility and obtain good image quality. Different age groups were given different doses of contrast agent, as shown in Table I. To observe the status of colonic involvement, the first administration of the contrast agent was administered 1.5–2 h before scanning. The dose was increased and patients received an enema in advance. Children with intestinal obstruction were not required to take the contrast agent due the good images produced by the large amount of liquid retention. In patients aged <5 years, isosmotic mannitol (2.5%) was administered via a nasogastric tube.

MRI examination was performed using a 1.5 Tesla scanner (Avanto; Siemens Medical Solutions, Erlangen, Germany) using an eight-channel phase array coil; the coronal and axial sections of the conventional MRI included coronal T2-weighted imaging (T2WI) with true fast imaging with steady state precession (TrueFISP), coronal and axial T1-weighted imaging (T1WI), T2WI with fat suppressed sequence and 3D volumetric interpolated breath-hold sequence (VIBE). Thereafter, the patients underwent an axial and coronal T1WI-enhanced scan using gadodiamide (0.2 ml/kg) as the contrast agent. Each sequence used a fat saturation technique. Patients were required to hold their breath several times during the VIBE sequence, while breathing was unaffected in the other sequences.

### MRI result analysis

After contrast, intestines with increased enhancement in adjacent walls were considered to be the involved lesions. The characteristics of the intestinal lesions, including the location, extent, wall thickness (>4 mm was considered as wall thickening and ≥1 cm was considered as severe thickening), signal intensity difference between lesions and adjacent normal intestine and parenteral complications (cellulitis, inflammatory mass, abscess and fistula and retroperitoneal or mesenteric lymph nodes enlargement) were assessed.

### VCE procedure

VCE was performed with an M2A capsule (PillCam SB; Given Imaging Ltd., Yoqneam, Israel) and the manufacturer’s software (RAPID) was used for the imaging analysis. In brief, this system consists of three major components: a miniature ingestible video capsule endoscope, data recording hardware and a workstation. Following activation and ingestion, the capsule endoscope acquired video images during natural peristaltic propulsion through the GI tract at a rate of two images per second. The capsule endoscope transmitted these images through radio waves to external sensor arrays and a data recorder was worn as a belt by the patient. Patients were instructed to fast overnight prior to VCE. Following activation and swallowing of the capsule, patients were allowed to drink liquids and eat solid food after 2 and 4 h, respectively. The data recording hardware was disconnected after 8 h and acquired data were downloaded. Patients were instructed to report natural excretion of the capsule in their stools.

### Interpretation

Results of MRE, VCE and CGR readouts were correlated with the final diagnosis based on the histopathological findings and on a combined endpoint of clinical, laboratory, conventional endoscopic, surgical and imaging findings.

### Statistical analysis

Statistical analysis of MRE, VCE and CGR findings for small bowel diseases was performed on a patient level. The sensitivity and specificity of these modalities were determined separately and then in conjunction with regard to the final diagnosis. Results were compared using the Fisher’s exact test (two-tailed). P<0.05 was considered to indicate a statistically significant difference. This test was selected based on a relatively small sample size.

## Results

### General information

All the children were able to drink the isotonic mannitol solution and successfully completed the MRI procedure without any discomfort or complications based on the combined diagnostic endpoint of all imaging, clinical, laboratory and histopathological findings. MRI findings (n=55) were compared with those of VCE (n=39) and CGR (n=31). Small bowel pathologies were identified in 55 patients in the study. VCE, MRE and CGR demonstrated sensitivities of 94.6, 85.7 and 71.1%; specificities of 72.7, 70 and 40%; accuracies of 89.6, 82.2 and 61.1%; positive predictive values of 92.1, 90.9 and 59.6%; and negative predictive values of 80, 58.3 and 40%, respectively. Results were compared using the Fisher’s exact test (two-tailed); assuming the absence of a statistical significance between VCE and MRE at P<0.05, there was a statistical significance between MRE and VGR. In three patients, VCE depicted mucosal pathologies missed by MRE, while MRE revealed 83 extraenteric findings in 55 patients. The dynamic evolution information of the gastrointestinal function provided by CGR is superior to that provided by MRE.

### MRE findings of the small intestine in Crohn’s disease

**Figure 1.**
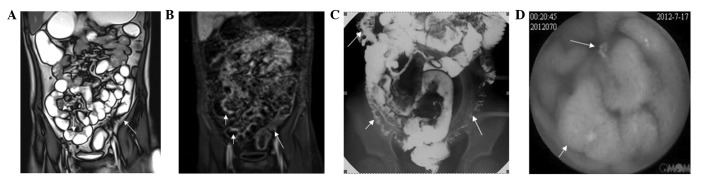
MRE, CGR and VCE correlation in a 14-year-old female patient with Crohn’s disease confirmed at histology. (A) Coronal T2-weighted TrueFISP image revealed that a dilated ileal loop with irregular wall thickening, increased mesenterial vascularity and separation of loops (white short arrow), ascending colon and descending colon wall thickening and luminal stenosis (white long arrow); (B) enhanced coronal T1-weighted image revealed wall thickening of the distal ileum (white short arrows) and descending colon (white long arrows) with significant enhancement; (C) the CGR image revealed distal ileum (white short arrows) and descending colon (white long arrows) luminal stenosis, mucosal fold thickening and edema, and broadening of fat space around the intestine; (D) VCE revealed ileum mucosal changes with ulcerations (white short arrow) and numerous small polypoid lesions (white long arrow). MRE, magnetic resonance enterography; CGR, conventional gastrointestinal radiography; VCE, video capsule endoscopy; TrueFISP, true fast imaging with steady-state precession.

**Figure 2.**
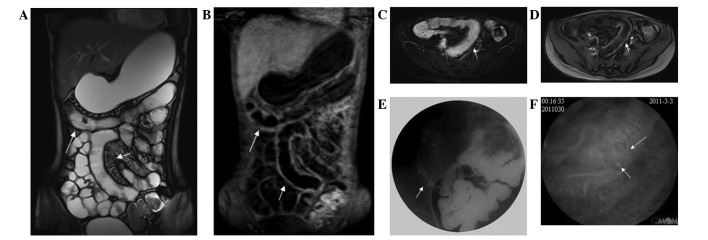
Correlation between MRE, CGR and VCE in a 16-year-old female patient with Crohn’s disease confirmed at histology. (A) Coronal T2-weighted TrueFISP image revealed that a dilated ileal loop with irregular wall thickening, increased mesenterial vascularity, separation of loops (white short arrow) and transverse colon wall thickening (white long arrow); (B) enhanced coronal T1-weighted image revealed wall thickening of the ileum (white short arrows) and transverse colon (white long arrows) with contrast enhancement; (C) axial T2-weighted fat suppression images revealed diffuse thickening and edema of the ileum (white short arrow); (D) enhanced axial T1-weighted image revealed diffuse thickening and edema of the ileum (white short arrow); (E) the CGR image revealed luminal stenosis of the distal ileum (short white arrows), mucosal fold thickening, edema and broadening of the fat space around the intestine; (F) VCE clearly revealed distal ileum mucosal changes with ulcerations (short white arrow), congestion and edema (long white arrow). MRE, magnetic resonance enterography; CGR, conventional gastrointestinal radiography; VCE, video capsule endoscopy; TrueFISP, true fast imaging with steady-state precession.

**Figure 3.**
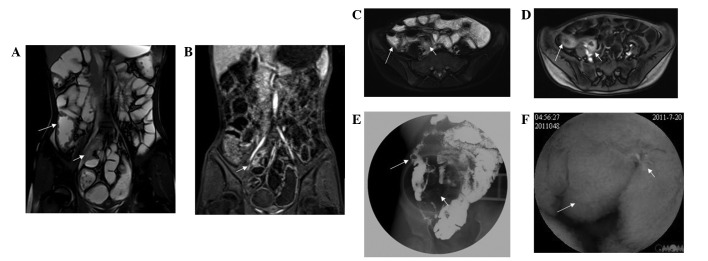
Correlation between MRE, CGR and VCE in a 15-year-old male patient with Crohn’s disease confirmed at histology. (A) Coronal T2-weighted TrueFISP image revealed irregular wall thickening and luminal stenosis of the distal ileum (short white arrow) and wall thickening of the ascending colon (white long arrow); (B) enhanced coronal T1-weighted image revealed wall thickening of the distal ileum (short white arrows) with contrast enhancement; (C) axial T2-weighted fat suppression images revealed wall thickening of the distal ileum (short white arrow) and ascending colon (long white arrow); (D) enhanced axial T1-weighted image revealed wall thickening of the ileum (short white arrow) and ascending colon (long white arrow) with contrast enhancement; (E) the CGR image revealed luminal stenosis of the distal ileum (short white arrows), mucosal fold thickening and edema; (F) VCE clearly revealed distal ileum mucosal changes with ulcerations, congestion, edema (short white arrow) and numerous polypoid lesions (long white arrow). MRE, magnetic resonance enterography; CGR, conventional gastrointestinal radiography; VCE, video capsule endoscopy; TrueFISP, true fast imaging with steady-state precession.

MRI clearly shows all cases with bowel disease. Among the 55 cases, 37 cases involved the small intestine (distal ileum) while 49 cases involved the colon, including the sigmoid colon, rectum, descending colon and ascending colon. An inflamed bowel was observed in a number of cases, with an average of 2.7 lesions per case. In these cases, enhanced MRI demonstrated a significantly augmented intestinal wall with an increased thickness of 5–14 mm. The majority of the lesions thickened circularly and unevenly, which narrowed the lumen (Fig. 1). Cellulitis or inflammatory masses were formed around the bowel presenting related mesenteric fat hyperplasia, fibrosis, thickening, short T1 high signal and significantly widened intestinal clearance (Figs. 2 and 3). Mesenteric lymph node enlargement and retroperitoneal lymph node enlargement were observed in three patients, while six cases presented ascites. Fistulae were not observed between the small intestine and colon; however, four cases with abdominal wall fistula and two cases with anal fistula were observed.

### Correlation between VCE and MRE

VCE and MRE findings were concordant and consistent with the final diagnosis in 33 cases. In six cases, MRE failed to depict limited mucosal disease, which was detected by VCE. In four patients, small bowel adhesions were diagnosed by MRE, whereas no abnormality was observed using VCE. MRE yielded additional information compared with VCE, not only for extraenteric processes, but also in defining the extent of mural disease. Although VCE alone demonstrated a higher diagnostic agreement compared with MRE, the Fisher’s exact test did not reveal a significant difference between the two modalities.

## Discussion

Previously, oral barium enema and small bowel air-barium enema were the main methods used in studying the intestinal mucosa and intestinal morphology of children with small bowel diseases. Given the length and bends of the small intestine, as well as the overlapping of tissues, simultaneous and overall demonstration of findings using conventional oral barium enema is not attained in a short time (12). VCE and MRE have emerged relatively recently and are increasingly utilized for small bowel assessment. Technical advances have enhanced the diagnostic capability of MRI in small bowel imaging and reports claim that MRI is becoming the method of choice for the evaluation of Crohn’s disease in certain reference centers, particularly in younger patients who may require repeated examinations (13). VCE is a unique tool, which allows direct visualization of the entire small bowel mucosa. Following its introduction to the clinical routine in 2001, VCE has introduced significant changes to the evaluation of obscure GI bleeding and has been widely established in the work-up algorithm for GI patients (14). A growing body of literature shows that VCE holds promise in the evaluation of diverse small bowel processes, including Crohn’s disease, tumors, graft-versus-host disease, inherited polyposis syndromes, celiac disease, radiation enteritis, abdominal pain and even unexplained diarrhea (15). In the current study, VCE was shown to be more sensitive than MRE in diagnosing small bowel pathologies and detecting limited mucosal pathologies in six patients, which were missed by MRE. The differences in diagnostic accuracy between the two modalities, however, lacked statistical significance, which was precluded by a small study population and selection bias with a low number of true negative cases.

Intestinal MRE imaging, which does not use ionizing radiation, reveals fine anatomical details of the intestine, provides functional and real-time information and allows direct observation of inflammatory changes in the small intestine. These inherent advantages make it well-suited in the assessment of small bowel diseases. Crohn’s disease is the primary indication for intestinal MRI examinations. Although oral barium CGR and CT enterography are able to observe and diagnose small bowel diseases, they use radiation, and so are not suitable for repeated examinations of children. One study demonstrated that MRE is extremely sensitive in discovering active Crohn’s disease with a higher detection rate compared with conventional oral barium enema and small bowel air-barium enema (16). MRI with a low spatial resolution does not show changes in the intestinal mucosa without contrast, while enhanced images identify early inflammatory changes in the intestinal wall (17). In addition, a study conducted by Siddiki *et al* demonstrated that no significant difference in sensitivity is observed between MRE and CT enterography (18). A study of Crohn’s disease confirmed the correlation of enhanced MRI findings, Crohn’s disease activity index and terminal ileum scope results (19). By analyzing the intestinal wall thickness and degree of enhancement, Malagò *et al* identified that the correlation between MRI assessments of disease activity and Crohn’s disease activity index was 91%, while the sensitivity and specificity of identifying terminal ileum inflammation were 81.8 and 100%, respectively (20). Moreover, MRE is better able to locate lesions. Given that Crohn’s disease may lead to intestinal obstruction, VCE is prohibited in this condition. MRE shows obstructed intestine stenosis or dilation, bowel aggregation and deformation (21). In the present study, MRE revealed Crohn’s disease in children with a sensitivity of 85.7, specificity of 70 and accuracy of 82.2%, respectively. Therefore, we consider MRE to have a high accuracy in the diagnosis of presence and location of small bowel Crohn’s disease, which is valuable in clinical practice. In addition, VCE is more expensive than MRE and it may not be suitable as a general means of examination based on a cost-benefit analysis.

The full expansion of the small bowel is a necessary condition for MRE. Two methods of dilating the small bowel (22,23) are the use of oral contrast agent (MR enterography) and duodenal intubation (MR enteroclysis). These methods were initially used in adults and are now improved and used in children with decent results (24). Although the enema provides a greater degree of expansion of the small intestine, its introduction to children has encountered challenges, which means the application is even more difficult. For the majority of children with inflammatory bowel disease, the inflamed bowel is expanded satisfactorily through oral MRE. Moreover, one study demonstrated that oral administration of contrast MRE exhibits similar sensitivity in detecting active inflammation in Crohn’s disease compared with enema MRE (24). We selected 2.5% isotonic mannitol as an oral MRE contrast since it is slightly sweet in taste, which is palatable to children. Furthermore, it is not absorbed by the alimentary system and it may be used in a large dose without affecting plasma osmolality. Combined with an abdominal breath navigation scan, clear constructed defects from respiratory movement and magnetic sensitiveness are not observed. The quality of the images is good. According to our study, large doses of oral isotonic mannitol satisfactorily filled the small bowel. MRE panoramically displays the lesion, including the lumen, the wall and the outer structure. Clinical doctors are able to interpret the images conveniently and more importantly, MRI without radiation reduces the risk for children and is therefore worth further application.

VCE and MRE have emerged relatively recently and are increasingly utilized for small bowel assessment. Technical advances have enhanced the diagnostic capability of MRI in small bowel imaging and studies have claimed that MRI is becoming the method of choice for the evaluation of Crohn’s disease in certain reference centers, particularly in younger patients who require repeated examinations (13). A meta-analysis that included 250 patients from nine studies investigated the role of VCE compared with a wide range diagnostic modalities, including barium radiography, CT enterography, MRE and colonoscopy with ileoscopy. The study demonstrated that VCE is superior in diagnosing reoccurrences of nonstricturing Crohn’s disease of the small bowel (25). By contrast, no significant differences were identified between VCE and the other modalities in the initial diagnosis of Crohn’s disease, although small sample sizes led to a type II error. Based on this analysis and on our own findings, larger study populations are required to establish the role of VCE in evaluating patients with a suspected initial presentation of Crohn’s disease.

The diagnosis of bleeding through capsule endoscopy has also been compared with that of GI barium imaging. A study by Kovanlikaya *et al* (26) examined the use of VCE in a total of 20 patients with a variety of small bowel diseases, including GI bleeding, inflammatory bowel disease, suspected sarcoma recurrence, functional abdominal pain, chronic diarrhea and small bowel polyps. Several procedures, including PE, gastroscopy, colonoscopy, angiography, intraoperative enteroscopy and radionuclide scanning, were performed. The diagnostic yield of VCE in the study by Kovanlikaya *et al* was 77.8%, while that of MRE was 75.0%. In a prospective study of 31 patients documented to have terminal ileal involvement with Crohn’s disease on colonoscopy, which included retrograde ileoscopy, the diagnostic yield of VCE was significantly superior to enteroclysis (89 vs. 37%; P>0.001) (24). VCE is a valuable development in the ongoing effort to effectively examine the small bowel. Compared with current technology, it is a relatively simple and well-tolerated procedure. VCE has proven to be superior to conventional techniques in the detection of obscure GI bleeding. In the present study, VCE and CGR demonstrated sensitivities of 94.6 and 71.1%; specificities of 72.7 and 40.0%; and accuracies of 89.6 and 61.1%, respectively. Therefore, VCE in small bowel Crohn’s disease is superior to CGR.

In summary, MRE is a simple, safe, non-invasive and effective method that does not require ionizing radiation in evaluating small bowel Crohn’s disease. VCE readily visualizes and characterizes subtle mucosal lesions missed by MRE, whereas MRE yields additional mural, perienteric and extraenteric information. Thus, VCE and MRE appear to be complementary methods. However, oral barium CGR and CT enterography are also able to identify and diagnose small bowel diseases. However, the two methods use radiation, which is not suitable for repeated examinations of children.

## Figures and Tables

**Table I.** Dose control of oral contrast agent in children according to age.

Age	Dose 45 min before scanning (ml)	Dose 15 min before scanning (ml)
<1 month	60–90	30–45
1 month -1 year	120–240	60–120
1–5 years	240–360	120–180
6–12 years	360–480	180–240
13–15 years	720	360
>15 years	720–960	360–480
